# Fibromatose desmoïde mammaire: à propos de deux cas et revue de la literature

**DOI:** 10.11604/pamj.2022.41.184.28549

**Published:** 2022-03-08

**Authors:** Ons Laakom, Haifa Bergaoui, Seifeddine Ben Hammouda, Abir Khalfalli, Leila Njim, Raja Faleh

**Affiliations:** 1 Service de Gynécologie du Centre de Maternité et Néonatologie de Monastir, 5000, Tunisie,; 2 Service d´Anatomie et Cytologie Pathologiques du CHU Fattouma Bourguiba Monastir, 5000, Tunisie,; 3 Service de Radiologie B du Centre de Maternité et Néonatologie de Monastir, 5000, Tunisie

**Keywords:** Fibromatose mammaire, imagerie, marges, traitement adjuvant, cas clinique, Breast fibromatosis, imaging, margins, adjuvant therapy, case report

## Abstract

La fibromatose mammaire est une tumeur mammaire bénigne d´origine mésenchymateuse, elle représente 0.2% des tumeurs du sein. Cet article relate deux observations de fibromatose mammaire étayant ses particularités diagnostiques, morphologiques, thérapeutiques et évolutives. Dans les deux cas, la tumeur mime sur le plan clinique et radiologique un cancer. La confirmation diagnostique est obtenue par une preuve histologique. La fibromatose mammaire se caractérise par une évolution locale et une tendance à la récidive, d´où l´intérêt de l´exérèse chirurgicale avec marges saines obtenues chez nos patientes. La place des traitements locorégionaux (la radiothérapie et la cryothérapie) et des traitements médicaux, en particulier les anti-estrogènes n´est pas clairement définie. En conclusion, la fibromatose mammaire doit être connue puisqu´elle mime en tout point un cancer de sein et se caractérise par un taux de récidive très important sans jamais donner de métastases.

## Abstract (en)

Breast fibromatosis is a benign breast tumor of mesenchymal origin, accounting for 0.2% of breast tumors. This study reports two cases of breast fibromatosis highlighting its diagnostic, morphological, therapeutic and evolutionary features. In both cases, this tumor clinically and radiologically mimicked a cancer. Diagnostic confirmation was based on histological examination. Breast fibromatosis was characterized by local progression and a tendency to recurrence, hence the role of surgical excision with free surgical margins in our patients. The role of locoregional treatments (radiotherapy and cryotherapy) and medical treatments, in particular anti-estrogen therapy, is not clearly defined. In conclusion, breast fibromatosis must be known as it mimicks breast cancer and is characterized by a very high recurrence rate, without ever developing metastases.

## Introduction

La fibromatose mammaire est une tumeur bénigne, mésenchymateuse, développée à partir des structures musculo-aponévrotiques. Il s´agit d´une entité tumorale rare et représente 0,2% des tumeurs mammaires primitives et 4% des fibromatoses extra-abdominales [1]. Cette pathologie mime sur le plan clinique et radiologique un cancer du sein. Seule l'histologie apportera le diagnostic [2]. L'exérèse chirurgicale de la tumeur avec des marges saines constitue le seul traitement. En cas d´exérèse incomplète, le risque de récidive tumorale locale est accru, cependant sans jamais donner de métastases [2].

## Patient et observation

### Premier cas clinique

**informations de la patiente**: madame SJ, une jeune fille de 17 ans, sans antécédents pathologiques notables, était suivie dans notre consultation pour une tuméfaction mammaire gauche depuis avril 2018.

**Résultats cliniques**: l´examen physique, montrait une masse de 3 cm, au niveau de l´UQS du sein gauche, douloureuse, ferme, mobile par rapport aux deux plans superficiel et profond et sans rétraction ni écoulement mamelonnaire.

**Figure 1:**
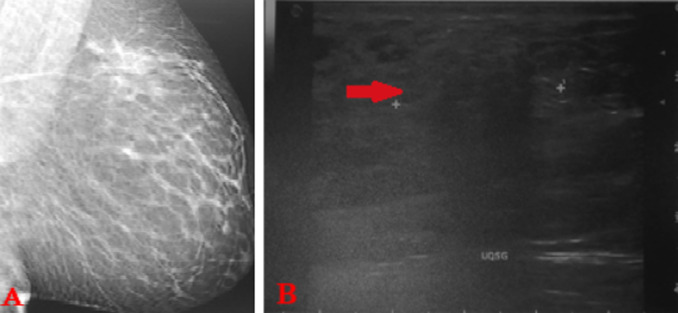
A) mammographie de profil du sein gauche; B) échographie mammaire gauche montrant une plage hétérogène de 4 cm mal limitée avec des zones hypoéchogènes atténuantes (flèche rouge), sans masse circonscrite siégeant à l´UQSG

**Figure 2:**
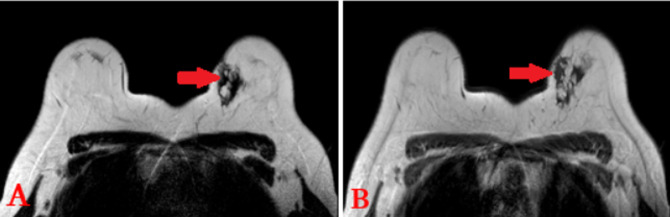
IRM mammaire en séquence pondérée en T1 et T2 (respectivement A et B); plage à l´UQSG de 41x16mm en discret hyposignal T1 et T2, mal limitée (flèche rouge)

**Figure 3:**
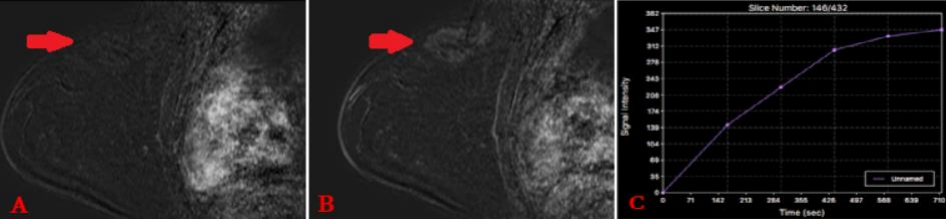
A et B) IRM mammaire en séquence pondérée T1 avec injection (phase précoce(A) et tardive (B)); C) IRM mammaire avec graphique en séquence de perfusion. Fibromatose mammaire sous forme d´une masse irrégulière à contours spiculés, prenant le contraste de manière rapide et progressive

**Evaluation diagnostique**: une mammographie a été faite et objectivant des seins de densité graisseuse type A de BIRADS avec asymétrie focale de densité du quadrant supérieur gauche. L´échographie mammaire a montré une masse hétérogène à l´UQSG étendue sur 4cm, nécessitant un complément d'exploration par IRM (Figure 1). L´imagérie par resonance magnétique (IRM) mammaire a mis en évidence une plage à l´UQSG de 4116 mm, superficielle, située à 18 mm du muscle grand pectoral, en discret hyposignal T1 et T2, mal limitée et prenant le contraste avec un rehaussement précoce avec un plateau (courbe type2). Le sein gauche a été classé ACR4 (Figure 2 et Figure 3). Une première micro-biopsie de la masse a révélé des importants remaniements fibreux du tissu mammaire de l´UQSG. La deuxième micro-biopsie a été composée de fragments de tissu adipeux sans structure glandulaire.

**Intervention thérapeutique**: étant donné la discordance clinico-histologique, une exérèse chirurgicale a été indiquée. Nous avons complété par une zonectomie gauche.

**Suivi et résultat**: le compte rendu anatomopathologique a conclu à une tumeur desmoïde mesurant 4 cm de grand axe, passant au contact des limites superficielles, supérieures et internes. Alors, nous avons repris la patiente pour faire des recoupes. L´examen anatomopathologique a conclu à des recoupes saines et le dossier a été présenté au staff multidisciplinaire où on a décidé de faire une surveillance rapprochée.

### Deuxième cas clinique

**informations de la patiente**: le deuxième cas est de madame B. N., âgée de 41 ans, sans antécédents pathologiques notables, quatrième pare qui était suivie pour un nodule du sein droit.

**Résultats cliniques**: l´examen initial objectivait un nodule de 10 mm au niveau du QSED, de consistances dures, mobiles et douloureuses à la palpation.

**Evaluation diagnostique**: on a complété par une écho-mammographie qui a montré une opacité stellaire en regard du nodule palpé. Le sein droit a été classé ACR4. Nous avons complété par la suite par une micro biopsie qui a montré une tumeur desmoïde du sein droit.

**Figure 4:**
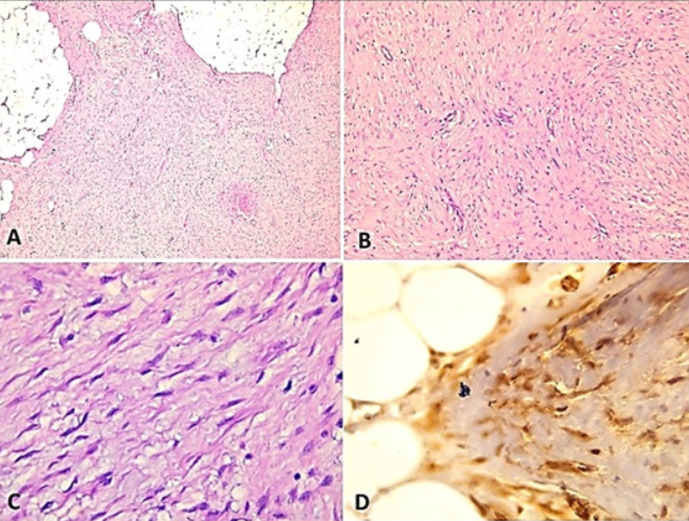
A) au faible grossissement: fibromatose desmoïde montrant histologiquement une prolifération mésenchymateuse mal limitée de cellularité variable (HE, A x40); B) au moyen grossissement; des zones d´aspect fibreux formés de cellules fusiformes disposées en faisceaux (HE, B x100); C) fort grossissement; des cellules avec un cytoplasme peu abondant et un noyau allongé d´allure fibroblastique et sans atypies. Les mitoses sont rares (1 mitose/10 champs au fort grossissement) (HE, Cx200); D) a l´immunohistochimie, les cellules tumorales montrent un marquage positif nucléaire à la bêta-caténine (HE, D x400)

**Intervention thérapeutique**: la patiente a alors bénéficié d´une tumorectomie du sein droit avec une recoupe profonde. L´examen anatomopathologique avait conclu à une tumeur desmoïde du sein droit mesurant 2x1,5cm. En effet, l´examen microscopique montrait une prolifération mésenchymateuse mal limitée de cellularité variable. Elle était formée des zones d´aspect tantôt lâche, tantôt fibreux. Les zones fibreuses sont faites de cellules fusiformes disposées en faisceaux. Ces cellules avaient un cytoplasme peu abondant et un noyau allongé d´allure fibroblastique et sans atypies. Les mitoses étaient rares (1 mitose/10 champs au fort grossissement) (Figure 4, A, B et C). La tumeur était focalement située au contact de la limite interne et à distance des autres limites latérales et des plans profond et superficiel. Les recoupes profondes et musculaires sont saines. A l´immunohistochimie, les cellules tumorales ont exprimé la bêta-caténine (Figure 4, D).

**Suivi et résultat**: le dossier était présenté au staff multidisciplinaire (gynécologie, carcinologie, radiothérapie…) et la patiente était proposée pour reprise et recoupe interne suivis d´un complément de radiothérapie. L´évolution était sans particularités, sans récidive tumorale.

## Discussion

L´étude des deux observations nous a permis de mieux approfondir nos connaissances sur la fibromatose mammaire puisqu´il s´agit d´une pathologie très rare et de mieux exploiter nos moyens de diagnostic (essentiellement l´IRM). La principale limite de notre travail est le nombre restreint de cas étudiés ce qui nous a empêchés de mieux étudier l´apport des traitements adjuvants. La fibromatose desmoïde du sein représente 0,2% des tumeurs mammaires primitives et 4% des fibromatoses extra abdominales [1]. Elle atteint des femmes âgées de 13 à 80 ans avec un pic de fréquence entre 30 et 50 ans [3]. Son origine est mal connue. Trois étiologies sont suspectées: origine traumatique, hormonale ou héréditaire. Dans certain cas, une composante génétique est présente; deux pourcent de ces tumeurs sont d´origine génétique, par mutation du gène adenomatous polyposis coli (APC), et entrent dans le cadre du syndrome de Gardner associé à la polypose adénomateuse familiale (PAF) [3]. Des cas sont décrits après un traumatisme, ou après chirurgie du sein comme pour les chirurgies de réduction mammaire [4,5], ou après prothèse mammaire [6,7]. Contrairement aux tumeurs desmoïdes abdominales, la fibromatose mammaire ne semble pas être associée à la grossesse [8]. La fibromatose mammaire est une tumeur bénigne du sein, mais sa présentation clinique, mammographique et échographique est souvent très suspecte [9,10].

Sur le plan clinique, la fibromatose mammaire se présente sous la forme d´un nodule ferme, mal limité, indolore, unilatéral, de taille variable (3 cm en moyenne), plus souvent périphérique que péri-aréolaire, parfois associé à une rétraction cutanée ou mamelonnaire. Cette lésion peut être douloureuse, surtout si elle infiltre les plans profonds. L´écoulement mamelonnaire est rare et les patientes n´ont pas d´adénopathie palpable. L´atteinte peut rarement être bilatérale [11]. L´imagerie prend souvent l´aspect d´une tumeur maligne. Les images observées sur la mammographie évoquent une lésion maligne (asymétrie de densité, opacité mal limitée à contours flous et irréguliers, spiculés). L´aspect mammographique le plus commun de la fibromatose est celui d´une masse spiculée non calcifiée, ce qui est observé chez la deuxième patiente de notre série. Des micro- ou macrocalcifications sont rares et correspondent à des lésions mammaires associées [11,12]. En échographie la fibromatose apparaît comme une masse solide, spiculée ou microlobulée, irrégulière, hypoéchogène avec une atténuation postérieure, mimant une lésion maligne [13]. L´imagerie par résonance magnétique a un intérêt pour apprécier un éventuel envahissement pariétal [7]. Bien que peu d´études aient été réalisées en IRM pour les fibromatoses, généralement, il s´agit d´une masse à contours mal limitées, souvent spiculée, iso-intense aux muscles, en iso/hypersignal T2 d´intensité variable [13], et hétérogène. La cinétique de rehaussement est variable, parfois d´allure bénigne progressive [14], parfois plus suspecte en plateau, comme dans notre premier cas, ou avec un wash out [5].

Le diagnostic préopératoire peut être évoqué à la microbiopsie mais le plus souvent, il est affirmé après une chirurgie diagnostique, ce qui est le cas chez notre première patiente. Sur le plan anatomopathologique, la fibromatose desmoïde se présente macroscopiquement comme une lésion assez bien circonscrite, de consistance indurée, fasciculée, nacrée et dont la taille varie de 5 à 10 cm de diamètre [15]. L´examen histologique montre une prolifération pauci cellulaire, à cellules fusiformes. Ces cellules sont d´allure fibroblastique et myofibroblastique, disposées en longs faisceaux parallèles au sein d´un fond riche en fibres collagène, souvent ondulés. Les cellules ne présentent habituellement pas d'anomalie nucléaire et les mitoses sont rares. La mauvaise limitation en périphérie avec infiltration du tissu mammaire adjacent en doigts de gant constitue un argument diagnostique important. Il s´y associe quelques vaisseaux, de petite taille et entourés souvent d'un espace clair. On note également, en périphérie, des raptus hémorragiques et des amas lymphoïdes [15]. L´étude immuno-histochimique montre une expression intense et diffuse de l'actine musculaire lisse et la bêta-caténine par les cellules tumorales. Les récepteurs estrogènes et à la progestérone ne sont pas détectables par immunohistochimie dans la fibromatose mammaire, comme son homologue extra mammaire [16]. Le diagnostic différentiel se pose surtout avec le carcinome métaplasique fibromatose-like, car ils partagent de nombreux similitudes: (1) Ils sont les deux infiltrants. (2) Les deux sont riches en collagène. (3) La cellularité tumorale est faible. (4) Les cellules ne sont pas aussi pléomorphes que les autres tumeurs [17].

Toutefois, il existe quelques caractéristiques morphologiques qui pourraient être utile à cet égard. Premièrement, le carcinome métaplasique fibromatose-like peut être associé avec un carcinome canalaire in situ ou à des amas de cellules épithélioïdes, tandis que cette association manque dans la fibromatose. Deuxièmement, les cellules dans la fibromatose desmoïde sont monotones avec des noyaux clairs et des nucléoles discrets, tandis que les cellules du carcinome métaplasique fibromatose-like ont des noyaux souvent hyperchromatiques avec un certain pléomorphisme. Troisièmement, des amas lymphoïdes ou même des follicules lymphoïdes sont souvent identifiés à la périphérie de la fibromatose, alors que le carcinome métaplasique fibromatose-like comporte habituellement un infiltrat inflammatoire chronique mélangés aux cellules fusiformes [15]. Un panel de marqueurs immuno-histochimiques utilisant des marqueurs épithéliaux comme la pan-cytokératine, la p63 et la cytokératine de haut poids moléculaire sera très utile afin de distinguer ces deux entités tumorales. En effet, le carcinome métaplasique fibromatose-like montre une positivité des cellules tumorales, même focale pour l'un de ces marqueurs [4]. La fibromatose mammaire est une tumeur localement agressive, mais sans potentiel métastatique. Le risque de récidive locale explique le caractère large de la chirurgie qui doit toujours consister en une tumorectomie élargie avec une marge de sécurité de 2 à 3 cm chaque fois que possible [11]. En effet, la fibromatose mammaire se caractérise par un taux élevé de récidive, entre 21 et 23 %. La plupart des récidives surviennent dans les trois ans mais un risque persiste jusqu´à dix ans [11,18]. Ce risque de récidive est d´autant plus élevé qu´il persiste une marge positive. Si les berges sont envahies, la reprise chirurgicale est indiquée, ce qui est le cas pour nos patientes [3]. En raison de l´importance du taux de récidive, l´association à de nombreux traitements adjuvants a été explorée.

Les traitements locorégionaux des tumeurs desmoïdes font partie de l'arsenal thérapeutique dans cette affection rare mais parfois sévère. La radiothérapie à la dose de 56 Gy peut être envisagée en cas de tumeur progressive malgré un traitement médical. Cependant, la littérature est peu abondante et hétérogène, tant par les modalités de l'irradiation (adjuvante vs récurrence) que par les techniques employées [19]. La cryothérapie est une technique en plein essor, qui peut être envisagée dans les mêmes situations cliniques, dans des équipes entraînées. Les résultats récents de cette technique de radiologie interventionnelle sont extrêmement prometteurs [19]. La place du traitement médical (les anti-inflammatoires, les anti-estrogènes, la chimiothérapie à faible dose…) n´est pas clairement établie en raison de la rareté de cette pathologie [20,21]. Il a probablement son indication en cas de récidive et de contre-indication à la chirurgie ou à la radiothérapie. En conclusion, les manifestations cliniques, radiologiques et histologiques, et les conduites thérapeutiques de nos deux observations étaient concordants avec la littérature. Il s´agit d´une pathologie très rare qui mérite d´être étudiée et connue en raison de sa similitude clinique et radiologique avec le cancer de sein. Le principal traitement est la chirurgie avec des marges saines puisque cette tumeur possède un potentiel élevé de récidive sans jamais donner de métastases. Les traitements adjuvants sont en cours d´évaluation vu le nombre restreint des patients atteints. Les deux patientes sont satisfaites des résultats et elles sont entrain de continuer leurs suivis dans notre consultation externe. Elles n´ont pas de récidive tumorale jusqu´à nos jours.

## Conclusion

La fibromatose mammaire est une pathologie très rare. Elle doit être connue car elle peut mimer, cliniquement et radiologiquement, un cancer. L´exérèse chirurgicale large avec des marges saines constitue le seul traitement de cette pathologie dont l´évolution est surtout caractérisée par la récidive locale. La radiothérapie est réservée aux cas de récidives locales lorsque la chirurgie est impossible.
